# Satiety, Taste and the Cephalic Phase: A Crossover Designed Pilot Study into Taste and Glucose Response

**DOI:** 10.3390/foods9111578

**Published:** 2020-10-30

**Authors:** Thanyathorn Sae iab, Robin Dando

**Affiliations:** Department of Food Science, Cornell University, Ithaca, NY 14853, USA; ts586@cornell.edu

**Keywords:** Taste, CPIR, sweeteners, satiety

## Abstract

The glycemic response produced by a food depends on both the glycemic index of the food itself, and on how the body reacts to the food as it is consumed and digested, in turn dependent on sensory cues. Research suggests that taste stimulation can induce the cephalic phase insulin response before food has reached the digestion, priming the body for an incoming glucose load. This glycemic response can consequently affect the amount of food consumed in a subsequent meal. The aim of this study was to investigate the effects on satiety of four preloads that differed in caloric content and sensory properties, in a small group of female and male participants (*n* = 10). Water, sucrose, sucralose, and maltodextrin were used to represent 4 different conditions of the preload, with or without energy, and with or without sweet taste. Individual plasma glucose concentrations were sampled at baseline, 45 min after consuming the preload, and after consuming an ad-libitum test meal. Hunger, fullness, desire to eat, and thoughts of food feeling were assessed every 15 min using visual analog scales. Results in male participants when comparing two solutions of equal caloric content, maltodextrin and sucrose, showed that plasma glucose concentration spiked in the absence of taste input (*p* = 0.011). Maltodextrin, while providing calories does not have the sweet taste that can serve to trigger cephalic phase insulin release to attenuate an incoming glucose load, and was accompanied by significantly greater change in feelings of satiety than with the other preloads. Despite the difference in postprandial blood glucose, the energy consumed in the test meal across the treatments was not significantly different in either males or females. Results highlight the importance of taste in stimulating the body for the efficient and effective glucose homeostasis.

## 1. Introduction

As obesity is linked to multiple health problems, including an increase in the risk of heart disease, type 2 diabetes, stroke, and certain cancers, it is important to understand the factors that control appetite and satiety. Energy intake is partially determined by the satiating properties of foods, which in turn are associated with protein, fat, and fiber content [1], sugar [2], food viscosity [3], volume [4], and in some circumstances, the resting blood glucose levels of the consumer [5]. The glycemic index (GI) of a food is a measure of the blood glucose raising potential of the food compared to a reference, pure glucose. The carbohydrate-content of foods is often classified into 3 groups; low GI foods such as soybean and apples, moderate GI foods such as bananas, and high GI foods such as white bread.

Consumption of high-GI foods can cause a spike in postprandial blood glucose, which when declining can drop below the initial baseline, promoting hunger, and encouraging overeating [6]. Conversely, the consumption of low-GI foods can lower fluctuation in blood glucose levels [7]. Thus, high GI diets are associated with behaviors that promote weight gain, where low GI diets have been proposed to affect a decrease in hunger and food intake, inducing satiety, and are recommended for weight management in type 2 diabetes patients [8,9].

In general, high blood glucose levels trigger insulin release to uptake glucose into muscles and adipose tissues [10]. This release of insulin can occur before food has reached the stomach, and thus before glucose from the meal has been liberated from foods to raise blood glucose levels, termed the cephalic-phase insulin release (CPIR). The CPIR can enhance our tolerance to glucose [11], preparing the body for an upcoming increase in plasma glucose content [12], and can be triggered by several modes of stimuli, including visual cues, food smell or taste, and the processes of mastication and swallowing [13].

Taste stimuli experienced as appetitive can stimulate ingestive motivation [14]. In humans and in rodents, when food is tasted, it induces the CPIR and increases plasma insulin levels. This mechanism occurs in rodents within the first five minutes of oral ingestion of sweet stimuli [11], acting through pathways including the T1R2+T1R3 taste receptors, the subunits that make up the sweet taste receptor [14] and a second pathway that seemingly does not require interaction with T1R3, as knockout mice that do not show an appetitive response to sucrose or glucose solutions still display CPIR responses [11].

A study from Just et al. [15] in humans confirmed that sweet taste arising from nutritive and non-nutritive sweeteners (sucrose and saccharin) activates the CPIR independent of an increase in blood glucose. Further, when isolating taste input through intragastric infusions of glucose in subjects tasting or not tasting during infusions, insulin was higher and blood glucose lower if sweetness was tasted while infusions took place, indicating that taste plays an important role in glucose homeostasis [16,17]. Taste is also linked to obesity, whereby the abundance of taste buds and taste papillae in both humans [18] and rodents [19] are reduced with weight gain.

Sucrose is a relatively high GI sweetener (although by no means the highest), which the body can easily digest, and is considered as the prototypic nutritive sweetener, providing 4 kcal of energy per gram. Even though the synthetic sweetener sucralose (branded as Splenda^®^, Heartland Food Products Group, Indianapolis, IN, USA) is marketed as being made from sucrose, by substituting 3 hydroxyl groups with chlorine our body cannot process it in the same manner as table sugar, to extract the chemical energy stored within. Therefore, sucralose provides negligible digestible calories and so is considered a non-nutritive sweetener [20], while still being experienced as sweet tasting. Digestible maltodextrins are short chain polymers of D-glucose units, and can be obtained by hydrolysis of edible starches. Maltodextrins have a high glycemic index, and can provide approximately 4 kcal per gram, similar to sucrose [21]. Maltodextrins, while caloric in nature, are experienced as almost tasteless, and thus if dosed at equivalent calories per gram to sucrose, can be employed as a tool to separate taste from caloric intake [22].

The objective of this study was to compare the effect of 4 preloads; water, maltodextrin, sucrose, and sucralose, on satiation, hunger and blood glucose response. Water provides neither taste stimulation nor glucose. Maltodextrin has no sweet taste but is a polymer of glucose molecules, which are readily digested. Sucrose provides both glucose and taste, and sucralose, taste but no digestible glucose. Thus, our study seeks to isolate the role of taste stimulation on the control of blood glucose, and subsequent feelings of hunger and satiety. Our hypothesis was that the sweet taste provided by sucrose would be vital in controlling feeding and feelings of hunger when compared to a preload providing glucose with no taste.

## 2. Materials and Methods

### 2.1. Subjects

The study was reviewed and approved by the Cornell University Institutional Review Board for Human Subject Research (protocol #1903008663). Ten healthy subjects (5 men, 5 women), 19–36 years old, with body mass index (BMI) from 19 to 37 kg/m^2^) were recruited from the Cornell sensory listserv. Participants were tested with over a 4 week period using RedJade sensory evaluation software (RedJade Sensory Solutions, LLC, Martinez, CA, USA), after a pre-screening questionnaire to select those fitting demographics and availability. Inclusion criteria included no self-reported smoking behavior, no reported food allergies, no aversion to artificial sweeteners, liking of the test food, acceptance of sucralose, not diabetic, and not currently seeking to control their weight. Additional panel demographics are provided in Supplemental Table S1.

### 2.2. Study Design

**Figure 1:**
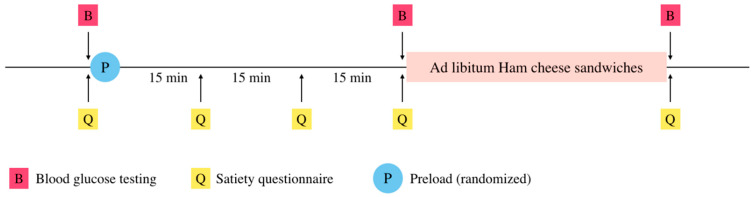
Schematic of study design on each test day.

Participants provided written consent, were asked to complete 4 sessions with at least 7-days between each test day, and were instructed to restrain from eating for 10 h prior to each test. During the fasting period, participants were only allowed to drink water, and were not precluded from cleaning their teeth on the morning of testing. Each session was conducted from 10 a.m. to 12 p.m. at the Cornell Sensory Evaluation Center. On each day of testing, individuals were given a 300 mL preload (see Figure 1), in a counterbalanced design. Five sets of questionnaires were given to rate hunger, fullness, desire to eat, and preoccupation with thought of food on visual analog scales (VAS) throughout the course of the study [23]. It was evident from the ratings of fullness that panelists may not have understood the scaling completely, as net change in fullness was not positive in several panelists after the test meal. This measure is presented for completeness, but nothing should be inferred from these results. Following the consumption of preload and first 4 questionnaires, an ad-libitum meal of toasted ham and cheese sandwiches was consumed, where participants were asked to eat until comfortably full. Small (5 oz) water cups were provided to avoid excessive water intake during the test meal. Finger stick capillary blood draws were performed by a licensed technician 3 times on each testing day; before and after the pre-load, and after test meal, to assay blood glucose level.

### 2.3. Pre-Loads

The four preload solutions used in the study were water, a moderately sweet sucrose solution (4.5% *w*/*v*: 54 kcal; Wholesale Grocers Inc., Keene, NH, USA), an equi-sweet (according to a study from Wiet & Beyts (1992) [24]) solution of the non-caloric sweetener sucralose (0.007% *w*/*v*: 0 kcal; VWR International, Radnor, PA, USA), and a solution of maltodextrin (4.68% *w*/*v*: 54 kcal; Eisen-Golden Laboratories, Dublin, CA, USA [25]) containing the same number of calories as the sucrose preload. All solutions were prepared one day ahead of testing and kept overnight in the refrigerator, then allowed to equilibrate to room temperature (20 °C) for at least an hour. Participants were also asked to rate the sweetness of each solution on the generalized labeled magnitude scale (gLMS), to confirm no sweetness from maltodextrin, and equal sweetness from sucrose and sucralose.

### 2.4. Test Meal

Participants should be familiar with the food served in a test meal, to avoid provoking a neophobic response resulting in reluctance to eat until satiation in the manner they usually would [26]. The test meal should also ideally be easily reproducible and have a reliable measure of energy intake [27], and be appropriate within the context of the timing of the meal, for example, breakfast food for a morning trial [28]. Therefore, toasted ham and cheese sandwiches were chosen as the test meal, as they are widely recognized for most people in the US, easily reproducible, and appropriate for the time of the study. Serving meals ad libitum means there should always be more food than participants can consume, to minimize the risk of plate cleaning, that may influence when a panelist ceases eating [29]. The sandwiches were served in an excessively large portion, toasted to ensure that participants did not deconstruct the samples, and panelists were instructed to refrain from selectively eating, for example leaving crusts unconsumed.

Toasted sandwiches were freshly made in the Sensory Evaluation kitchen during each day of the study. Each sandwich consisted of two sliced of Stroehmann^®^ King white bread (Stroehmann Bakeries, L.C., Horsham Township, PA, USA), two Kraft^®^ Singles American cheeses (Kraft Heinz Food Company, Chicago, IL, USA), and one slice of Great Value^®^ water added cooked ham (Wal-Mart Stores, Inc., Bentonville, AR, USA). This provided approximately 2.37 kilocalories per gram. Each sandwich was cut into four pieces. Approximately 500 g of sandwiches were served to each panelist, on an 8 × 8 × 15/8 inch aluminum tray, with panelists monitored, and another tray provided if the panelist came close to finishing the first. Total food consumed to fullness was recorded by weighing the trays before and after the meal.

### 2.5. Statistics

Sweetness intensities of the four preloads were analyzed with one-way repeated measures ANOVAs and post hoc Tukey’s tests for normally distributed data, and Friedman’s tests for non-normal, using GraphPad Prism 5 (GraphPad Software, San Diego, CA, USA), while the relationship between preloads, plasma glucose concentration, energy consumed, and VAS ratings of satiety were analyzed using 2-way ANOVAs with sex as a term in the analysis, and using a linear mixed model built with SPSS 26 statistical analysis software (IBM, Armonk, NY, USA), where effects tested below *p* < 0.05 were considered statistically significant. Each rating related to satiety (hunger, fullness, desire to eat, and thoughts of food) was subtracted from baseline ratings at the start of the test to represent change in these ratings as the test progressed.

## 3. Results

### 3.1. Maltodextrin Preload Were Perceived No Sweeter than Water, Sucralose as Sweet as Sucrose

**Figure 2:**
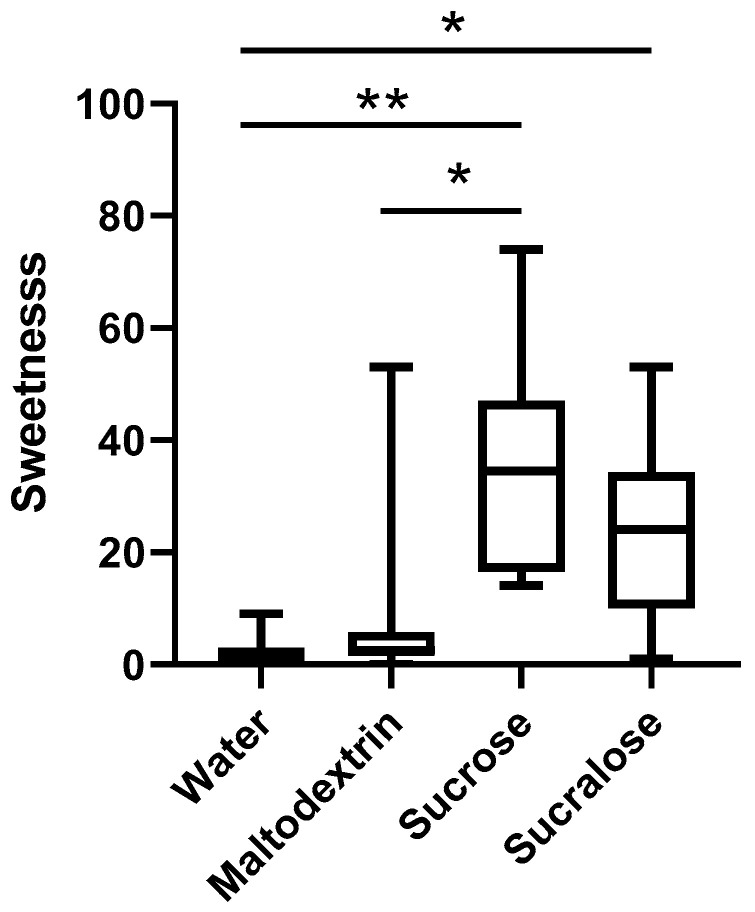
Sweetness intensity of water, maltodextrin, sucrose and sucralose, measured on the generalized labeled magnitude scale (gLMS). Box represents median, upper and lower quartiles, whiskers show min and max. Stars denote statistical significance, * *p* < 0.05; ** *p* < 0.01.

All preload solutions were tested for sweetness, using the gLMS. The sweetness of water versus maltodextrin was not significantly different (*p* = 0.668; Figure 2). For sucrose and sucralose solutions, there was also no statistically difference between these two solutions (*p* = 0.342), although sucrose was sweeter than both water (*p* = 0.003) and maltodextrin (*p* = 0.037), and sucralose was sweeter than water and maltodextrin, but only significantly sweeter than water (*p* = 0.010). Thus we can say that sucrose and sucralose were no sweeter than one another, nor were water and maltodextrin.

### 3.2. Consuming Caloric Preload Did Not Reduce Energy Consumption in Test Meal

**Figure 3:**
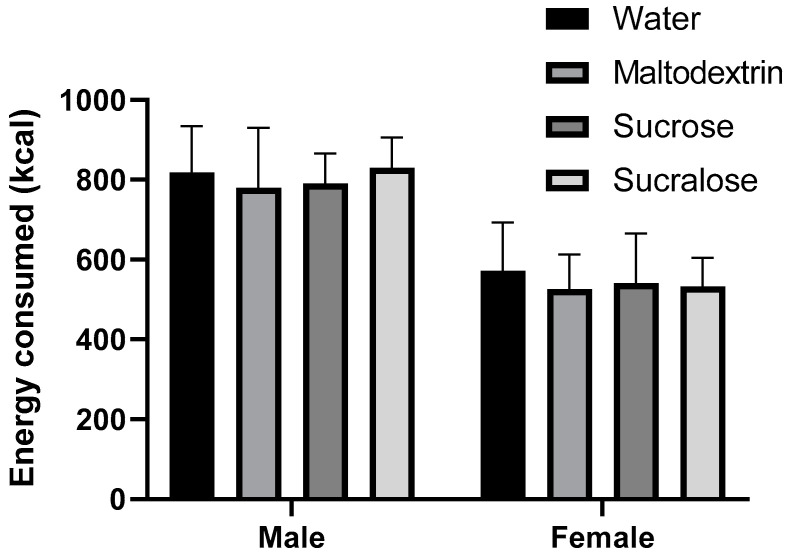
Energy consumed (kcal) during the test meal in male and female panelists. Bars represent mean plus SEM.

As unsurprisingly men consumed a significantly greater number of calories than women, and male versus female responses to foods have been noted to differ considerably [30], results were separated by sex. Within sex, no significant difference was found between how much food was consumed following any preload, in either male or female panelists (Figure 3). While we also tested the effect of body weight on energy consumption, in our panel neither body weight (*p* = 0.777), nor the interaction between body weight and preload (*p* = 0.826) were significant predictors of energy consumption. Finally, variation in macronutrient intake were also tested, with no differences between treatments evident (see Supplemental Table S2, Supplemental Figure S1).

### 3.3. Plasma Glucose Concentration Spiked after Tasteless Maltodextrin Preload in Males, but Not after an Equal Amount of Calories from Sucrose

**Figure 4:**
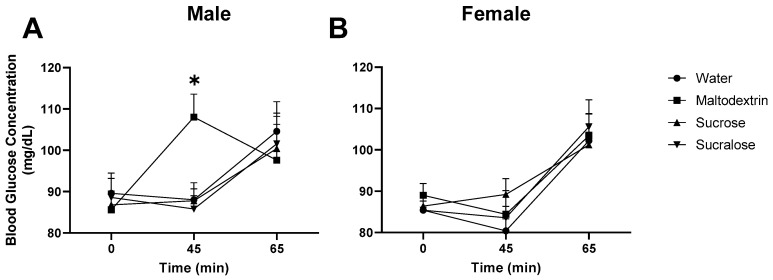
Blood glucose concentration (mg/dL) at baseline (0 min), after the pre load (45 min), and after the test meal (65 min) for water, maltodextrin, sucrose and sucralose preloads, in male (**A**) and female (**B**) panelists. Bars represent mean plus SEM. Stars denote statistical significance, * *p* < 0.05.

Both time and the interaction between time and preload had a significant effect on blood glucose concentration in males. In analyzing each time point individually (Figure 4A), both preload (*p* = 0.016, 2-way repeated measures ANOVA) and the interaction between sex and preload (*p* = 0.007) significantly differed in the testing, with differences seemingly more evident in males than females. A significant increase in blood glucose occurred after the preload for maltodextrin, when compared to sucrose (*p* = 0.011, Friedman’s test with post-hoc Dunn’s multiple comparisons test). The maltodextrin preload represents a liberation of glucose into the bloodstream without the protective effects of sweet taste triggering the CPIR. No significant difference in glucose response to any of the preloads was found in females (Figure 4B).

### 3.4. Satiety Ratings in Males Reflect Trends from Blood Glucose Measurement

**Figure 5:**
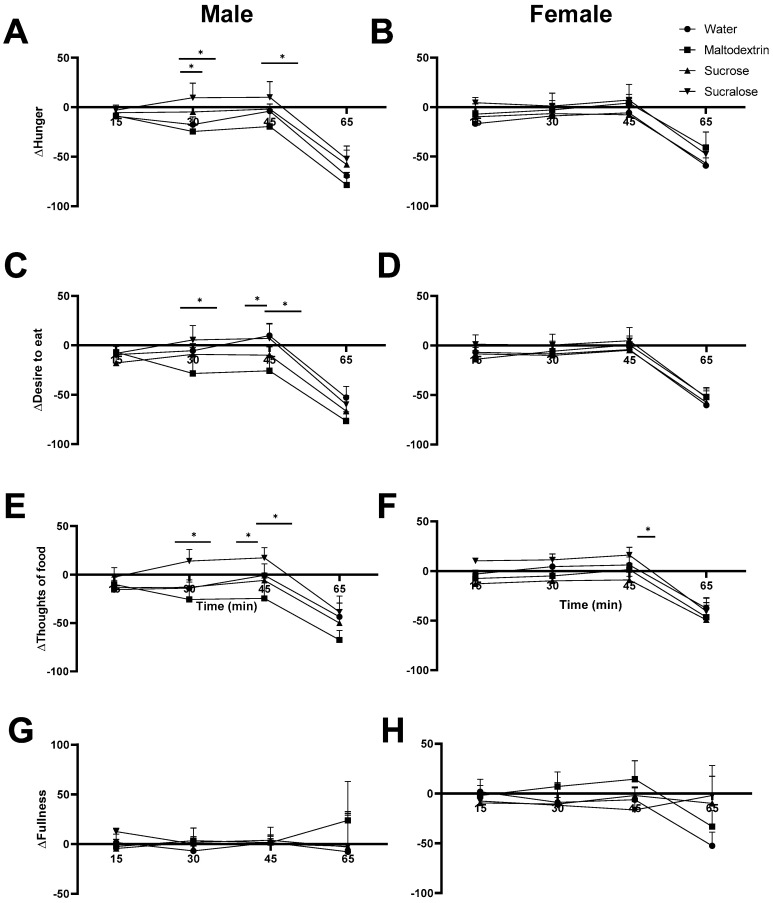
Difference from baseline (Δ) of hunger, desire to eat, thoughts of food and fullness in male (**A**,**C**,**E**,**G**) and female (**B**,**D**,**F**,**H**) panelists respectively. Bars represent mean plus SEM. Stars denote statistical significance, * *p* < 0.05.

Significant differences in satiety ratings between the preloads were evident again in male participants, with differences less apparent in females, where blood glucose did not seem to vary (Figure 5). After maltodextrin consumption, male subjects’ change in hunger, desire to eat, and preoccupation with thoughts of food significantly differed from those after other preloads, both at the 30-min and at the 45-min time point. Results were in general agreement with data on blood glucose, whereby the maltodextrin preload triggered a blood glucose spike not present after the other preloads. In general, results seemed to support that a change in satiety that would usually be accompanied by a blood sugar increase such as that after a meal occurred earlier with the maltodextrin preload, where no taste input acted as a prelude to the bolus of glucose the body received, although this did not affect amount of food eaten in the test meal.

## 4. Discussion

### 4.1. Plasma Glucose Is Influenced by Cephalic Phase Responses

Men and women’s blood glucose concentration responded to the preloads in a notably different manner (Figure 4). At 45 min after maltodextrin ingestion, plasma glucose concentration in males spiked, but this was not observed in females, which may be due to differences in body compositions in men and women. A study from Geer and Shen pointed out that men and women have different amounts of adipose tissue and sex hormones, where men have more visceral and hepatic fat and women have more peripheral and subcutaneous fat. The higher amount of hepatic and visceral adipose tissue in men has been linked to higher insulin resistance, with women more sensitive to insulin partially due to estrogen [31]. When insulin resistant, the body responds less to an elevation of insulin, impairing the body’s ability to take up glucose [32]. It is possible that males in our study were more resistant than females to insulin released through cephalic phase responses other than taste (the act of swallowing, mouthfeel, visual cues), resulting in a more prominent blood glucose spike after maltodextrin.

Water, as a control solution, did not provide calories or sweet taste, thus should have elicited minimal CPIR. Within the first 45-min from water preload, after the preload but before subjects consumed the test meal, plasma glucose concentration remained stable, as seen in Figure 4. Sucrose provided both calories and sweet taste, which should elicit the CPIR, thus attenuating an increase in blood glucose arising from sucrose. Just et al. (2008) reported that an increase in plasma insulin was found within the first 5 min after oral stimulation from sucrose [15]. In this study, blood glucose sampling was at 45 min after ingestion, and thus represents a snapshot, where both glucose and insulin have affected blood glucose. Results are reminiscent of those from a similar crossover designed study from Smeets et al. (2005), where 5 male panelists were given water, glucose, aspartame or maltodextrin [33]. In agreement with our hypothesis, an early insulin spike was observed with glucose, but not with maltodextrin. No difference in calories consumed was evident for sucrose versus water, or for any other preload. In a study by Anderson and Woodend (2003), sucrose reduced the amount consumed in a subsequent meal. Both a low (25 g sucrose/300 mL) and a high dose (135 g sucrose/300 mL) reduced subsequent meal intake and suppressed hunger [34]. In our study, we used a 4.5% *w*/*v* sucrose solution, which was around half of the lowest concentration in Anderson and Woodend’s study. It is possible that any effect of sucrose on satiety was too small to measure in our study. The sucralose solution provided sweet taste but not calories. The sweet taste from sucralose may still elicit a CPIR response to cause an insulin spike [35], but without digestible glucose, sucralose should not have a significant effect on the postprandial glycemia [36]. In this study, we did not see any distinct drop in blood glucose after either sweet solution. This may also be explained by our infrequent blood sampling time, which may have missed the specific time when glucose dropped.

### 4.2. Tasteless Versus Sweet-Tasting Preloads

Maltodextrin provided calories but no sweet taste. Maltodextrin is a starch derived product, consisting of a number of D-glucose molecules, but should not directly elicit the CPIR from taste, instead having to be digested to release glucose by amylases and alpha-glucosidases, leading to an insulin response later in consumption [37]. In this study, we found a blood glucose spike in male panelists after subjects consumed maltodextrin, but not after water, sucrose or sucralose. Participants also reported larger drops in hunger, desire to eat, and thoughts of food after receiving maltodextrin. This indicates that the increase in blood glucose elicited by maltodextrin may have led to reduced hunger with this treatment. Blood glucose is the best known biomarker for hunger [38]. Another possible explanation could lie in the natural properties of maltodextrin as a thickening agent, which forms gels and creates viscosity in solutions [37]. Maltodextrin is widely used in the food industry as a bulking agent, stabilizer, or thickening agent [39]. In this study, maltodextrin may also have provided a slightly thicker mouthfeel, promoting a feeling of greater satiety than the other preloads. Evidence suggests that increasing the viscosity or texture of a solution may lead to increased feelings [40,41,42] or expectations [43,44] of satiety, or lead to lower consumption to reach the same satiety [45]. Zijlstra et al. (2008) found that subjects consumed more when receiving low viscosity liquid, and less when receiving high viscosity liquid, likely due to a slower eating rate [3]. Another study reported that consuming high viscosity food led to a slower eating rate and a delay of gastric emptying, with lower hunger and desire to eat [46]. Nonetheless, the only results we are aware of suggesting that viscosity can affect blood glucose levels concern varying viscous fiber constituents to alter thickness [47,48], thus our interpretation remains the most likely.

The mean rating of maltodextrin’s sweetness were slightly higher than water, although not significantly, which may be explained by the dumping effect. Dumping is when a restricted response is offered in a questionnaire, causing people to report their feeling on a sensation on an inappropriate scale, even when attributes are not related to one another [49]. In this case, we only asked participants to rate the sweetness of the preloads, without any other attributes provided to rate. It is possible that subjects may have detected that the preload was something other than water (for example a slight viscosity change), but since there was only sweetness to report on the ballot, subjects may have dumped their ratings into the sweetness scale. Nonetheless, the effects recorded were negligible.

### 4.3. Energy Consumption

Though serum glucose concentration was differentially affected by the preloads, the energy consumed to satiate male or female panelists was not significantly different among the four solutions. This result is in agreement with a study from Kendall et al. (2018), which investigated the effects of sucrose and isomaltulose, that differentially affect glycemic response, on subsequent meal intake. Results showed that subsequent energy intake and satiety did not vary significantly, compared to differing plasma glucose concentrations [5]. This result is in line with multiple reports noting a lack of caloric compensation after sweetener consumption, such as with aspartame versus sucrose sweetened lemonade, served during, or 30 or 60 min before, a test meal [50], water versus low or high aspartame beverages one hour before a test meal [51], or treatments with water, aspartame or encapsulated aspartame [52], none of which resulted in changes in energy intake, or caloric compensation after consumption of these sweeteners.

There were several limitations in this study that should be noted. First, we tested only ten panelists in this study, which does not represent a large sampling, and may have influenced our interpretation. Secondly, blood was not drawn as frequently as in some studies, so fine details on the temporal response were also not assessed. Finally, a direct measure of plasma insulin would have further strengthened the conclusions that could be drawn in this set of experiments. Future studies addressing these issues may elucidate more details on this topic.

## 5. Conclusions

Sweet taste acts on taste receptors in the mouth, which are linked to cephalic phase insulin release. Maltodextrin does not provide taste to elicit the CPIR, while still being readily digested into glucose. Our results suggest maltodextrin produced a blood glucose spike after consuming the preload in male participants, which occurred alongside an increased change in ratings of satiety. Despite these results, average energy consumption during the test meal was similar between the preloads. Our results further support an important role for taste input in glucose homeostasis and satiety.

## Supplementary Materials

The following are available online at https://www.mdpi.com/2304-8158/9/11/1578/s1, Figure S1: Macronutrient Comparison, Table S1: Demographic Information, Table S2: Macronutrient details.
