# Supplementary material for: Satiety, Taste and the Cephalic Phase: A Crossover Designed Pilot Study into Taste and Glucose Response
Source: Foods. 2020 Oct 30;9(11):1578. doi: 10.3390/foods9111578 (PMC7693382; doi:10.3390/foods9111578)
Supplement: Supplementary file 1 [file foods-09-01578-s001.zip › Supplemental table 1.docx]

Table 1. Male and Female (n=10)

Demographic Categories Frequency Percentage

Gender

Male 5 50%

Female 5 50%

Age

15-19 1 10%

20-24 4 40%

25-29 3 30%

30-34 0 0%

35-39 2 20%

Ethnicity

White or Caucasian 1 10%

Hispanic or Latino 3 30%

Black or African-American 1 10%

Asian or Pacific Islander 4 40%

Other* 1 10%

BMI

Underweight (<18.5) 0 0%

Normal weight (18.5-24.9) 5 50%

Overweight (25-29.9) 4 40%

Obesity (>30) 1 10%

Smoking Behavior

Yes 0 0%

No 10 100%

*White/Asian

Table 2. Male (n=5)

Demographic Categories Frequency Percentage

Age

15-19 0 0%

20-24 2 40%

25-29 2 40%

30-34 0 0%

35-39 1 20%

Ethnicity

White or Caucasian 0 0%

Hispanic or Latino 2 40%

Black or African-American 1 20%

Asian or Pacific Islander 1 20%

Other* 1 20%

BMI

Underweight (<18.5) 0 0%

Normal weight (18.5-24.9) 0 0%

Overweight (25-29.9) 4 80%

Obesity (>30) 1 20%

Smoking Behavior

Yes 0 0%

No 5 100%

*White/Asian

Table 3. Female (n=5)

Demographic Categories Frequency Percentage

Age

15-19 1 20%

20-24 2 40%

25-29 1 20%

30-34 0 0%

35-39 1 20%

Ethnicity

White or Caucasian 1 20%

Hispanic or Latino 1 20%

Black or African-American 0 0%

Asian or Pacific Islander 3 60%

Other* 0 0%

BMI

Underweight (<18.5) 0 0%

Normal weight (18.5-24.9) 5 100%

Overweight (25-29.9) 0 0%

Obesity (>30) 0 0%

Smoking Behavior

Yes 0 0%

No 5 100%

*White/Asian
