# Supplementary figures and images for: Satiety, Taste and the Cephalic Phase: A Crossover Designed Pilot Study into Taste and Glucose Response
Source: Foods. 2020 Oct 30;9(11):1578. doi: 10.3390/foods9111578 (PMC7693382; doi:10.3390/foods9111578)

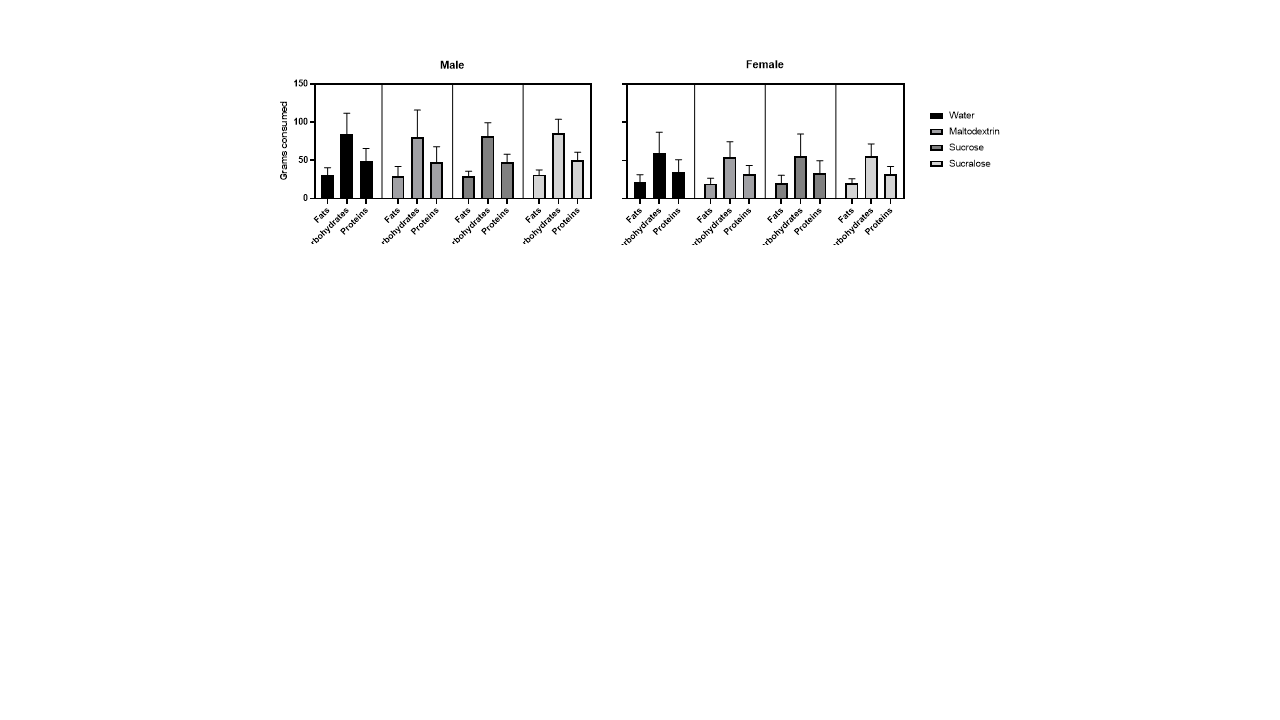

Supplement: Supplementary file 1 [file foods-09-01578-s001.zip › Supp Figure 1.tif]
